# Deposition of CdSe Nanocrystals in Highly Porous SiO_2_ Matrices—In Situ Growth vs. Infiltration Methods

**DOI:** 10.3390/ma17174379

**Published:** 2024-09-05

**Authors:** Raktim Baruah, Munira Dilshad, Marco Diegel, Jan Dellith, Jonathan Plentz, Andreas Undisz, Adriana Szeghalmi, Maria Wächtler

**Affiliations:** 1 Department of Chemistry and State Research Center OPTIMAS, RPTU Kaiserslautern-Landau, 67663 Kaiserslautern, Germany; 2 Leibniz Institute of Photonic Technology, 07745 Jena, Germany; 3 Institute of Materials Science and Engineering, Chemnitz University of Technology, 09125 Chemnitz, Germany; 4 Otto Schott Institute of Material Research, Metallic Materials, Friedrich Schiller University, 07743 Jena, Germany; 5 Institute of Applied Physics, Friedrich Schiller University Jena, 07745 Jena, Germany; 6 Fraunhofer Institute for Applied Optics and Precision Engineering, 07745 Jena, Germany

**Keywords:** CdSe quantum dot, thin film, porous silica

## Abstract

Embedding quantum dots into porous matrices is a very beneficial approach for generating hybrid nanostructures with unique properties. In this contribution we explore strategies to dope nanoporous SiO_2_ thin films made by atomic layer deposition and selective wet chemical etching with precise control over pore size with CdSe quantum dots. Two distinct strategies were employed for quantum dot deposition: in situ growth of CdSe nanocrystals within the porous matrix via successive ionic layer adsorption reaction, and infiltration of pre-synthesized quantum dots. To address the impact of pore size, layers with 10 nm and 30 nm maximum pore diameter were used as the matrix. Our results show that though small pores are potentially accessible for the in situ approach, this strategy lacks controllability over the nanocrystal quality and size distribution. To dope layers with high-quality quantum dots with well-defined size distribution and optical properties, infiltration of preformed quantum dots is much more promising. It was observed that due to higher pore volume, 30 nm porous silica shows higher loading after treatment than the 10 nm porous silica matrix. This can be related to a better accessibility of the pores with higher pore size. The amount of infiltrated quantum dots can be influenced via drop-casting of additional solvents on a pre-drop-casted porous matrix as well as via varying the soaking time of a porous matrix in a quantum dot solution. Luminescent quantum dots deposited via this strategy keep their luminescent properties, and the resulting thin films with immobilized quantum dots are suited for integration into optoelectronic devices.

## 1. Introduction

Semiconductor nanocrystals (NCs) have emerged as the pivotal material in nanotechnology for the applications in optoelectronics as well as sensing and biomedical imaging, owing to their unique size-dependent optoelectronic properties [1]. Among them, quantum dots (QDs) stand out for their excellent and wide-ranging optical and electronic properties, which can be adjusted by varying their size. As the size of the QD is reduced, discrete quantized energy levels are observed in contrast to the continuous energy band structure observed in bulk materials [2,3]. This phenomenon is a consequence of the strong spatial confinement of electron and hole motion when the QD size is below the Bohr radius [2,3]. One of the most extensively studied systems comprises cadmium selenide (CdSe) QDs. CdSe QDs exhibit narrow photoluminescence spectra and can be designed to show high photoluminescence quantum yields (PLQYs); they are therefore highly suitable for device applications such as in light-emitting diodes (LEDs) [4,5,6] or in sensing [7,8] applications. The tunability of electronic properties via size allows for the optimization of the valence band and conduction band energy levels allowing emission color, or ensures sufficient driving force for the energy and electron transfer processes forming the basis for sensing [7,8,9] concepts or the application of QDs as light absorbers to drive photocatalytic reactions [10,11,12]. The synthesis of high-quality (with respect to crystallinity and control over size distribution) CdSe QDs is achieved via the well-established hot injection method [13,14]. QDs resulting from this synthetic approach are usually capped with long-chain aliphatic surface ligands, e.g., tri-octylphosphine oxide, octadecylphosphonic acid, oleic acid, hexadecylamine, etc. [15]. The surface ligands provide colloidal stability, stabilize the surface of the QDs, and saturate dangling bonds, which are the source of surface trap states or introduce additional trapping states, depending on the anchoring functional group of the surface ligand [16,17,18]. Surface functionalization can also alter the dispersibility of the QDs in different solvents, rendering them suitable for applications in various solvent environments, e.g., in aqueous environments for sensing in biological systems [7] or photocatalytic applications [12,19] in aqueous environments. 

The colloidal solutions of the QDs have the advantage to allow for solution processing, e.g., to generate thin films with controlled homogeneity and packing density, enabling fine tuning of optoelectronic properties and allowing for much more flexibility and large-scale processing compared to methods such as chemical vapor deposition, epitaxial growth, etc. [20,21]. For many applications, dispersed NCs have to be transferred into thin film architectures, e.g., for application in LEDs [22,23], materials for photovoltaic devices [24,25,26], photoelectrode materials for photocatalytic applications [27] and sensors [28], or optoelectronic devices [29] for detection of radiation. Beyond thin film production by deposition of particles on substrates, immobilization and thin film production by integration of QDs into porous matrices, e.g., mesoporous silica, is a very interesting approach. The porous matrices support ordered assembly controlled by the structure of the porous matrix [30] or can be used to control size [31] via pore sizes of the matrix serving as a template. The porous matrix surrounding can shield the QDs from environmental factors such as oxygen and moisture and improve light extraction [32], reducing thermal effects and leading to improving long-term stability and efficiency, as observed, e.g., for LED devices [31,33,34]. Furthermore, porous matrices can support the targeted function by reduction of non-radiative recombination, contributing to improved quantum efficiency and brightness of the LEDs [35], or support charge carrier separation by co-immobilizing donors and acceptors, reducing charge carrier transfer distances and enhancing photocatalytic performances [36]. Furthermore, it can facilitate efficient diffusion of reactants and products, leading to improved reaction rates and yields in photocatalytic processes in the confined surrounding of the pores [37].

Two general strategies exist to deposit QDs inside a porous matrix. For a direct generation of NCs within the porous material, the successive ionic layer adsorption and reaction (SILAR) approach can be used [30,38,39]. For example, Besson et al. [30] and Wang et al. [39] demonstrate the growth of CdS QDs and CdSe QDs, respectively, in porous silica matrices via in situ growth of QDs inside the pores. Short immersion times and repeated cycles allow for control over loading and particle sizes, though with limited precision and broad size distributions [30]. To overcome the missing precision in controlling size and size distribution of NCs generated via the SILAR method within the porous matrices, infiltration of pre-synthesized particles employing methods with high control over size distributions can be employed. This approach has been used to deposit colloidal QDs inside mesoporous silica particles in the so-called “wet mixing method” [33,34,40], but also has been adapted to porous silica layers by soaking in a solution of dispersed QDs [41].

In this contribution, we investigate strategies for the incorporation of nanocrystalline CdSe into porous silica layers with pore diameters in the 10 s of nm range produced via atomic layer deposition (ALD) [42]. Fabrication methods based on ALD are shown to be advantageous with respect to control over thickness of the porous layer and rigidity of the porous structure compared to sol-gel methods [43,44,45]. Two general strategies are employed to deposit nanocrystalline CdSe into the porous material: in situ growth of CdSe NCs using the SILAR approach and infiltration of pre-synthesized QDs. Analysis of the structure and optical properties of the thin films with dependence on experimental parameters, e.g., pore size and deposition times, is performed to evaluate the quality of the produced layers. 

## 2. Materials and Methods

For the NC synthesis, the following chemicals were used: Tri-octylphosphine oxide (TOPO, 99%), Trioctylphosphine (TOP, 97%), Cadmium oxide (CdO, 99.99%), Cadmium acetate hydrate (Cd (OAc)_2_·xH_2_O, 99.99%), Selenium (Se, 99.99%), Sodium selenide (Na_2_Se, 95%), Toluene (99.8% anhydrous) and Methanol (99.8% anhydrous), purchased from Sigma Aldrich, and Octadecylphosphonic acid (ODPA, 97%), purchased from Carl Roth (Karlsruhe, Germany).

CdSe QDs were synthesized following established protocols [46,47]. A total of 60 mg of cadmium oxide (CdO), 0.28 g of octadecylphosphine oxide (ODPA), and 3.0 g of trioctylphosphine oxide (TOPO) were mixed in a 25 mL three-neck flask. The mixture was heated to 80 °C under a N_2_ atmosphere until melting under stirring. A vacuum was applied to remove traces of water, and after bubble formation stopped, the mixture was heated to 150 °C and evacuated for 1 h. Then, under N_2_ flow, the reaction mixture was heated up, and at around 300 °C the solution became optically clear and colorless. At 320 °C, 1.5 g of trioctylphosphine (TOP) was injected into the solution. When the temperature reached 380 °C, a solution of TOP–Se (0.058 g Se dissolved in 0.360 g TOP) was injected, and the temperature was kept at 370 °C for 5 min. Then, the reaction mixture was cooled down by removing the heating mantle, and when the temperature reached 60 °C, 10 mL toluene was injected. After the synthesis, QDs were precipitated by adding 10 mL MeOH to the reaction mixture, with centrifugation at 5300 rpm. The precipitate was redispersed in toluene, and was repeatedly precipitated and redispersed for cleaning three more times. Finally, the QDs were redispersed in 10 mL of toluene and stored inside a glove box (N_2_ atmosphere).

Nanoporous SiO_2_ layers were prepared using the methods reported by Ghazaryan et al. [42] Briefly, a heterostructure of SiO_2_–Al_2_O_3_ was deposited by atomic layer deposition (ALD) using a sequence of 2 cycles of SiO_2_ and 3 or 4 cycles of Al_2_O_3_. The sequence was repeated 230 and 330 times, respectively. The growth rate of the SiO_2_ and Al_2_O_3_ was about 0.1 nm/cycle. The film thickness of the [2:3]x230 sample was 123 nm, with 230 nm for the [2:4]x330 sample, as estimated by spectroscopic ellipsometry measurements. After deposition, the Al_2_O_3_ was selectively etched in H_3_PO_4_ (85%) solution, and a porous SiO_2_ matrix was formed. The maximum pore size as estimated in a previous work by SEM was 10 nm and 30 nm, being smaller when less Al_2_O_3_ was removed from the SiO_2_ matrix [42]. Porous silica layers were prepared both on fused silica to enable optical characterization and on silicon substrates to perform electron microscopy (Plano Gmbh, Wetzlar, Germany) for imaging.

QDs were grown in situ in the pores of the silica matrix by the Successive Ionic Layer Adsorption Reaction (SILAR) method at room temperature following a protocol described by Sankapal et al. [48] The porous silica layer (either on fused silica or silicon substrate) was immersed first into a Cd (OAc)_2_ solution (40 mL Cd (OAc)_2_ in methanol, 5 µM) for 5 min. After taking it out, the layer was rinsed with methanol and dried under vacuum. Then, the layer was immersed into a Na_2_Se solution (40 mL Na_2_Se in methanol, 6 µM) for 5 min, followed by rinsing with methanol and drying under vacuum. This completed one cycle of immersion. The immersion procedure was repeated several times, and layers were produced by applying 5, 10, 15, and 20 immersion cycles.

To infiltrate pre-synthesized QDs into porous silica, two strategies were applied—drop-casting of a QD solution on porous silica layers and immersion and soaking of porous silica layers in a QD solution. For drop-casting, 100 µL of QD solution (3 µM, toluene) was dropped on porous silica layers. Once the solvent was completely evaporated, the layers were rinsed with toluene to remove QDs just adsorbed at the surface and dried under vacuum. For some of the layers, after the initial drop-casting step using a solution of QDs, an additional amount of solvent was dropped on the substrates to wash QDs just sitting on the surface into the pores. Alternatively, the porous silica layers were immersed in 3 mL of QD solution (0.4 µM, toluene) to soak QDs into the pores. Soaking times of 0.5 h, 2 h, and 24 h, followed by rinsing with toluene and drying under vacuum, were applied. For comparison and to prove infiltration into the porous structure, a normal glass substrate, cleaned by washing with acetone, methanol, Hellmanex, and water, was drop-casted with 100 µL of QD solution (3 µM, toluene), and one other normal glass substrate was immersed in a QD solution (0.4 µM, toluene) for 24 h followed by washing with toluene. 

UV–vis absorption spectroscopy in the wavelength range from 200 nm to 1000 nm was performed using a Jasco V-780 spectrophotometer (Jasco, Hachioji, Tokyo, Japan) . Colloidal dispersions were measured in a 1 cm quartz cuvette, and for thin film measurements a special film holder was used. Absorption spectra measured in thin films contained strong wavelength-dependent scattering contributions, which were corrected via fitting a polynomial background [49] as described in Figure S1. 

The photoluminescence spectra of the QD solution were recorded in a 1 cm cuvette using a FLS 980 Edinburgh Instruments (Livingston, UK) Fluorimeter upon excitation at 400 nm. The photoluminescence spectra of the porous silica layers on fused silica were recorded using a Horiba Fluorolog-3 (Glasgow, Scotland). The excitation wavelength was set to 400 nm. 

To determine the photoluminescence lifetimes, Time Correlated Single Photon Counting (TCSPC) was performed with a Horiba DeltaFlex spectrometer (Glasgow, Scotland) with a pulsed NanoLED (peak wavelength of 389 nm, pulse duration of 1.3 ns) from Horiba. For this, QD solutions were prepared in a 1 cm quartz cuvette. QD thin films and porous layers were measured using a suitable thin film holder.

Transmission Electron Microscopy (TEM) images were recorded using a JEM-ARM200F NEOARM (Jeol) (Jeol, Akishima, Tokyo, Japan) operating at 80 kV. For this, Colloidal QDs were deposited on a carbon-coated Cu grid (purchased from PLANO GmbH, Wetzlar, Germany). To evaluate the average size and size distribution of the particles, the images were processed using an ImageJ 1.53a program [50].

Scanning Electron Microscopy (SEM) images of QDs deposited on silicon wafers and porous silica on silicon wafers were recorded using a JEOL JSM-6700F scanning electron microscope (Jeol, Akishima, Tokyo, Japan) . Additionally, cross-sectional images of the porous silica layers were collected after breaking the substrate and depositing a carbon layer.

For elemental analysis, Energy Dispersive X-ray Spectrometry (EDX) on porous silica layers on silicon wafers using a Bruker silicon drift detector SDD-5030 (Brucker Corporation, Billerica, MA, USA) (30 mm^2^ detector area) with 10 keV electron energy was performed. Grazing incidence X-ray diffraction (GIXRD) on porous silica layers on fused silica was performed using a PANalytical X’Pert Pro MPD (Malvern Panalytical, Malvern, Worcestershire, UK) ((Cu-Kα radiation, 1.541 Å) with an omega angle of 2°, a 2theta range from 10° to 90°, a 0.026° step size, and measuring times from 1 h to 15 h). 

Secondary Ion Mass Spectrometry (SIMS) was performed using a Hiden Analytical SIMS Workstation equipped with 5 keV Cesium and Oxygen ion sources for ionization and sputtering for the material within a spot of 50 µm and a high-transmission quadrupole secondary ion mass spectrometer (Hiden Analytical Ltd., Westbrook, Warrington, UK) . A layer of less than 10 nm Pt was deposited on top for electrically conductive surfaces to prevent electrical charging during measurement.

## 3. Results and Discussion

Porous silica layers were prepared using atomic layer deposition (ALD) of a heterostructure of SiO_2_:Al_2_O_3_, followed by selective etching of Al_2_O_3_ [42]. In order to systematically investigate the extent of QD deposition by applying different methods, porous layers with two different pore sizes were prepared. Top view and cross section SEM images of the porous layers are shown in Figure S3. Maximum pore sizes of 10 nm and 30 nm were determined from SEM images. The thicknesses of the layers are in the range from 123 nm and 230 nm. 

The first preparation route applied was to grow CdSe NCs directly inside the porous matrix. For this, a SILAR protocol was adopted [48]. Methanolic solutions of Cd (OAc)_2_ as the Cd^2+^ source and Na_2_Se as the Se^2−^ source were used. A 5 min immersion of the porous layer into the Cd (OAc_2_) solution led to a monolayer adsorption of Cd^2+^ ions attached to the porous surface via Van der Waals and electrostatic forces. Loosely bound Cd^2+^ ions were removed by rinsing the porous layers before immersion into a Na_2_Se solution, which initiated the reaction of Cd^2+^ and Se^2−^ to form CdSe. Further successive cycles of immersions of the porous layers into Cd (OAc)_2_ and Na_2_Se solutions led to successive growth of CdSe NCs. To observe the growth in the successive immersion steps, samples were prepared with 5, 10, 15, and 20 immersions for both the layers with 10 nm and 30 nm pore size.

**Figure 1:**
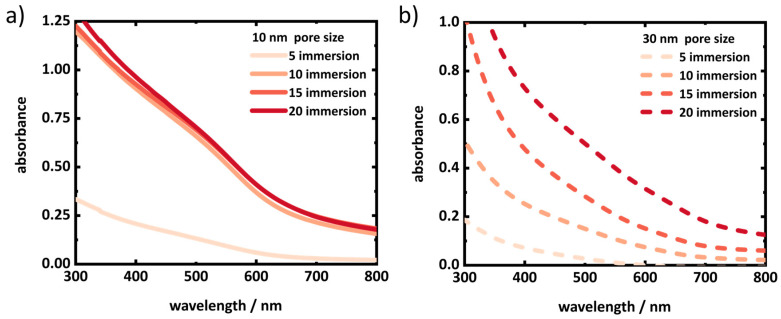
Absorption spectra of porous silica layers on fused silica substrates of (**a**) 10 nm and (**b**) 30 nm pore size with different numbers of SILAR immersion cycles. The absorption spectra of the thin films contain strong wavelength-dependent scattering contributions. A correction for this scattering contribution, as suggested in the supporting information Figure S1, could not be performed reliably for these samples, because it was not possible to assign the region of no absorption, due to the broad absorption feature, in the investigated spectral range.

Steady-state UV–vis absorption spectroscopy was performed on the porous silica layers, which shows absorption features below 700 nm (Figure 1). With the number of immersion steps, the absorption feature increases in intensity, and the onset of absorption shifts to higher wavelengths. This indicates, on the one hand, an increased loading of the SiO_2_ matrix with CdSe, and on the other hand, an increase of the crystal size of the CdSe deposited (Figure 1). Similar behavior was observed upon in situ growth of CdS NCs on TiO_2_ via the SILAR method [48]. Typically, CdSe QDs exhibit distinctive electronic transitions from the valence band to the conduction band due to the presence of quantized energy levels at the band edges [51]. In contrast, the absorption spectra show only very broad features, without any distinct peak. This can be related to a broad size distribution caused by uncontrolled growth of CdSe crystals and aggregation of smaller NCs, with an increasing number of immersion cycles forming larger particles. The absorption features of the layers with 30 nm pore size exhibit a notable increase in absorbance with the number of immersion cycles. In contrast, the absorption of the layers with 10 nm pore size show a pronounced rise from the initial immersions to 10 cycles, followed by a saturation. This suggests that the limited pore volume of 10 nm pore-sized layers is fully occupied by the NCs or that narrow parts of the porous structure are clogged, preventing further deposition of CdSe, while the 30 nm pore-sized layers continue to fill. No photoluminescence was detected from the layers. This potentially is due to low crystal quality, i.e., low crystallinity and a high density of the NCs grown via this method. 

**Figure 2:**
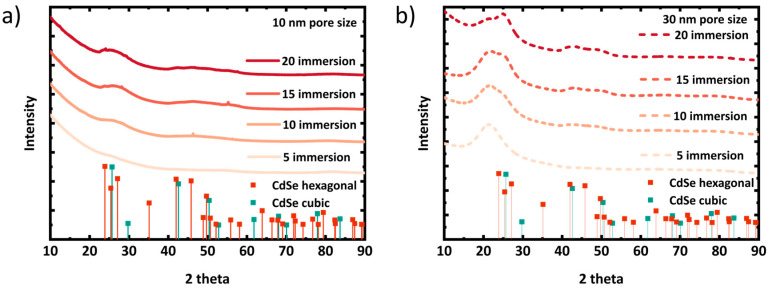
XRD pattern of porous silica layers (on fused silica substrate): (**a**) 10 nm and (**b**) 30 nm pore size with different numbers of SILAR immersion steps. The XRD patterns shown in the figure are without any background correction.

To evaluate the crystal quality, the samples were characterized by GIXRD. Figure 2 shows the GIXRD patterns from porous silica layers with in situ grown CdSe QDs. As the CdSe crystallites grown inside 10 nm and 30 nm pores are very small in size, the corresponding XRD peaks are broadened but seem to grow in and slightly sharpen with increasing immersion cycles [52]. The broad and less intense XRD peaks of CdSe NCs make it very challenging to distinguish between the hexagonal phases. However, conducting the in situ growth at room temperature increases the likelihood of cubic crystal phase formation. From a thermodynamic perspective, the cubic phase is more stable at lower temperatures, while the hexagonal phase is more stable at higher temperatures [52]. It seems, at least from the dataset of the sample with the 30 nm pore size, that with the number of immersions, XRD peaks evolve, with the potentially growing size of the embedded crystallites representing a cubic pattern with its characteristic 2theta peaks at 24.84° (111) and 42.83˚ (220) (ICSD: 620421). Nevertheless, this conclusion needs to be regarded with care. On the other hand, due to smaller pore volume, CdSe QDs grown in 10 nm pores seem to be smaller than in 30 nm pores, indicated by even broader XRD peaks than the layers of the 30 nm pore size.

**Figure 3:**
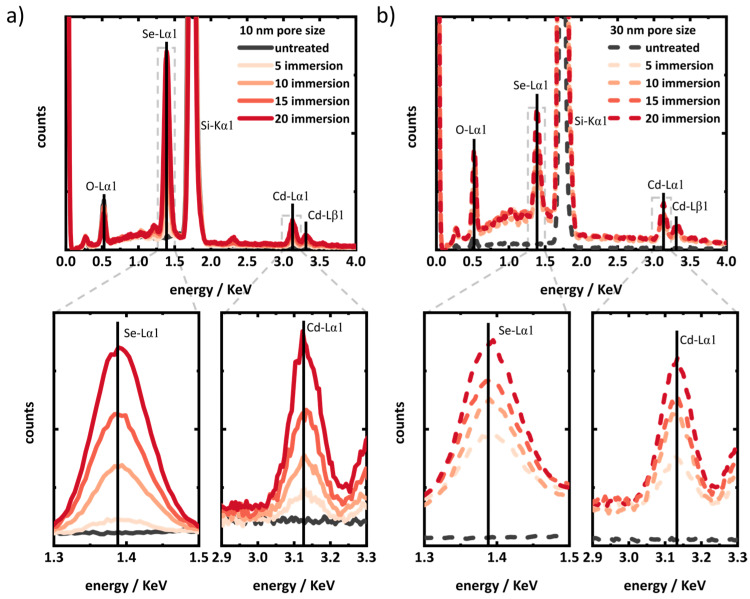
EDX spectra of porous silica layers (Si wafers) of (**a**) 10 nm and (**b**) 30 nm pore size with different SILAR immersions. Respective bottom panels show the zoomed regions of Se and Cd peaks.

Further, to observe the CdSe QDs in the porous matrices, Energy Dispersive X-ray Spectrometry (EDX) was performed. Figure 3 depicts the EDX spectra of 10 nm and 30 nm porous layers. While collecting the EDX spectra, an area of 200 µm^2^ was exposed to the electron beam. In comparison to spot analysis, this large area of exposure gives a good comparability among different samples, lowering the danger of local overestimation. For both the 10 nm and 30 nm pore-sized layers, there is a relative increase in the intensities of the Cd and Se peaks with the number of immersion cycles (Figure 3). The increasing Cd and Se peak intensities reflect the increasing amount of Cd and Se deposited in the layers with every SILAR immersion cycle. 

**Figure 4:**
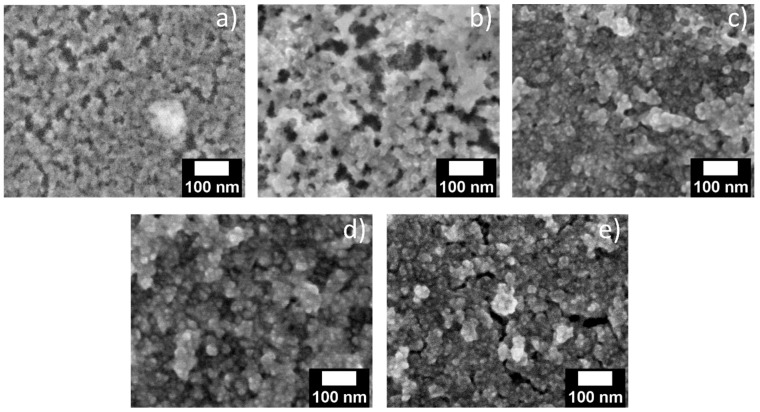
SEM images of (**a**) untreated porous silica layers of 30 nm pore size in silicon wafer and with (**b**) 5, (**c**) 10, (**d**) 15, and (**e**) 20 SILAR immersions.

SEM images of porous layers in silicon wafers were collected to observe the local structure of CdSe NCs in the porous structure. Figure 4 and Figure S4 depict the SEM images of 30 nm and 10 nm pore-sized layers, respectively. Because the resolution of SEM is not sufficient to image single NCs, which might be even more complex in the case of a broad size distribution, a clear presence of CdSe NCs cannot be confirmed from the top view image. Nevertheless, it appears from the top view images that the pores gradually fill with an increasing number of immersion cycles. 

To summarize, the in situ SILAR growth leads to the deposition of CdSe NCs on the porous matrix. Steady-state absorption and photoluminescence spectroscopy, as well as XRD and EDX analysis complemented by SEM imaging, indicates that the NCs are probably deposited within the porous network. However, the deposition at the surface cannot be ruled out and prevented. The NCs exhibit indications of a cubic phase with low crystallinity and a broad size distribution. Additionally, they demonstrate an absence of photoluminescence, which is likely related to the high number of trap states, which in turn decreases the PLQY. For any optoelectronic applications, it is essential to embed particles with better controllable properties, such as distinct electronic transitions, controllable size, and high crystalline NCs that support high PLQYs. The synthesis of highly crystalline CdSe NCs with a narrow size distribution via the SILAR method is a challenging process, necessitating the exploration of alternative strategies. These strategies must be capable of embedding NCs with high crystallinity and controllable properties, including a narrow size distribution. This leads us to seek an alternative porous silica and CdSe QD ensemble that exhibits superior performance, including a high PLQY, good crystallinity, and a narrow size distribution.

To achieve this goal, we employed an alternative method, which allowed us to benefit from the well-controlled properties of colloidal QDs synthesized via hot injection methods performed at high temperatures (usually > 300 °C), resulting in CdSe QDs with precise control over size and size distribution, high crystallinity, high PLQY, narrow photoluminescence band width, etc. [14]. However, such synthesis routes are typically not applicable for embedding CdSe QDs directly during synthesis into porous silica layers due to the lack of thermal stability of the silica matrix in that temperature range [53]. Additionally, the crystal growth in the hot injection method undergoes a fast nucleation mechanism [14], which is hindered by the porous matrix, resulting in inefficient growth inside the pores. As this rules out the feasibility of employing the hot injection method for direct growth within the porous layer, we explore the possibility of deposition and infiltration of pre-synthesized QDs into the porous silica matrix. 

**Figure 5:**
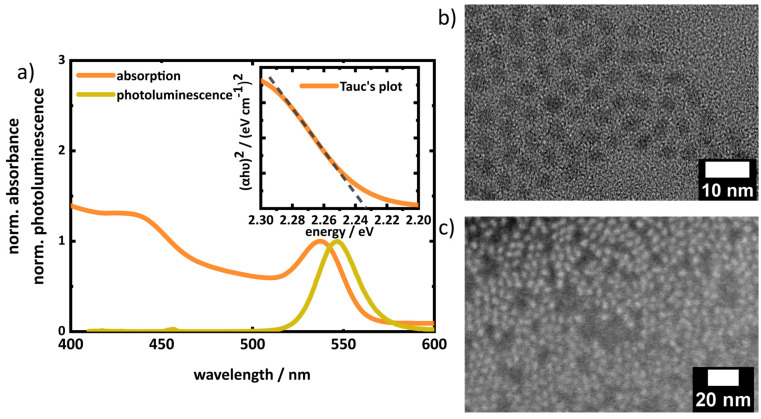
(**a**) Normalized absorption and photoluminescence spectra (λ_ex_ 400 nm) of the CdSe QDs with 2.9 nm diameter in toluene and Tauc’s plot for band gap calculation (inset). (**b**) TEM and (**c**) SEM image (on silicon wafer) of 2.9 nm CdSe QDs used in this study.

For our study, CdSe QDs were pre-synthesized via hot injection (see experimental) [46,47]. The QDs were majorly covered with TOPO as the surface ligand, along with TOP and ODPA (used with Se and Cd precursors in the QD synthesis), which made them dispersible in non-polar solvents like toluene, chloroform, hexane, etc. The absorption spectra of the pure QD dispersion (toluene) shows the characteristic electronic transition from the valence band to conduction band levels (Figure 5a). The feature at 537 nm corresponds to the lowest excitonic band edge 1S_(e)_-1S_3/2(h)_ transition [51]. A band gap of the QDs of 2.2 ± 0.04 eV was derived from Tauc’s plot, which is in agreement with the band edge photoluminescence peak position (547 nm, 2.26 eV) [54]. The absolute PLQY is 3.9%. The diameter of the QD was estimated from the spectral position of the lowest excitonic transition in the absorption spectra by the empirical formula derived by Yu et al. [55] to approximately 2.9 nm, in good agreement with the average diameter determined by statistical analysis of TEM images (3.2 ± 0.5 nm, Figure 5b and Figure S5a,b). Furthermore, a thin film was prepared by drop-casting the QDs on a silicon wafer to visualize the QDs in layers via SEM (Figure 5c).

Pre-synthesized CdSe QDs were infiltrated into porous silica of pore size 10 nm and 30 nm via (a) drop-casting of a QD solution (3 µM) in toluene on the porous silica layers and (b) soaking porous silica layers in a QD solution (0.4 µM) in toluene. We started with a simple drop-casting followed by washing the layers with toluene to remove QDs sitting just on top of the surface. To induce higher infiltration of QDs, an additional amount of solvent (toluene) was dropped on the layers after the first step of drop-casting followed by washing the layer in toluene. In the soaking method, porous layers were immersed in a QD solution. The loading of QDs was influenced by soaking time. Because of the surface ligand TOPO, QDs are hydrophobic. Therefore, in each washing step after infiltration, the QDs sitting on the hydrophilic porous layer surface should be effectively removed.

**Figure 6:**
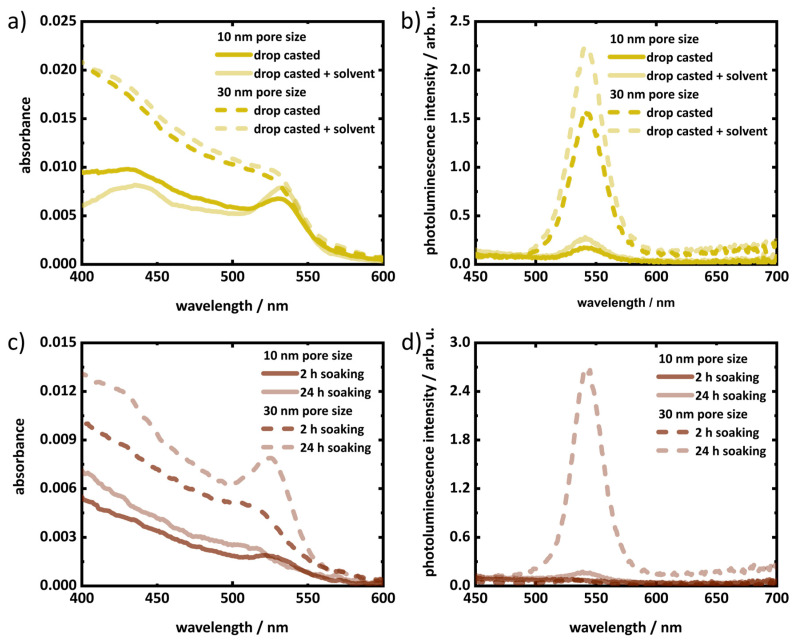
Absorption spectra (**a**) and photoluminescence spectra (**b**) of 10 nm (solid lines) and 30 nm (dashed line) porous layers treated by drop-casting and drop-casted followed by solvent treatment. Absorbance spectra (**c**) and photoluminescence spectra (**d**) of 10 nm (solid lines) and 30 nm (dashed line) porous layers with 2 h and 24 h soaking. The wavelength scattering correction as described in Figure S1 was employed in the absorption spectra (**a**,**c**).

The deposition of pre-synthesized QDs on porous silica layers was confirmed by steady-state UV–vis absorption spectroscopy and photoluminescence spectroscopy. Figure 6 and Figure S6 show the spectra of porous silica layers after QD infiltration. The absorption spectra were corrected for background signal from the substrate and scattering contributions from the porous layer (see Supplementary Information Figure S1). The characteristic feature of the lowest band edge transition (1S_(e)_-1S_3/2(h)_) is clearly visible in the absorption spectra of the treated layers, which confirms the presence of QDs. After QD infiltration via both drop-casting and soaking, for the porous layers with 30 nm pore size a higher absorbance is observed than for the layer with 10 nm pore size. This is an indication of a higher loading of QDs in bigger pores (i.e., 30 nm) than in 10 nm pores, which indicates an easier accessibility of the larger pores for the QDs. For the drop-casting routine, treatment with additional solvent resulted in a relative increase compared to the drop-casted layers without additional solvent treatment (Figure 6a). For the soaking strategy, the amount of QDs infiltrated into the pores increases with time of soaking. The gradual increase of the absorption of QDs in porous layers for soaking times 0.5 h to 2 h (Figure S6a) and 2 h to 24 h (Figure 6c) indicate the increase of the amount of infiltrated QDs. The band edge photoluminescence peak of the QDs at 541 nm in the photoluminescence spectra of the porous layers further confirms the presence of QDs in the treated porous layers (Figure 6b,d and Figure S6b). Due to the inhomogeneous distribution of QDs and strong scattering contributions, quantitative comparison of both absorbance and photoluminescence intensity among samples is not possible. It can still be assumed that due to higher loading of QDs, porous silica layers of 30 nm pore size show higher photoluminescence intensity than the layer of 10 nm pore size for all infiltration methods (Figure 6 and Figure S6). Similar to the absorption spectra, additional drop-casting of toluene increases the photoluminescence intensity due to higher infiltration of QDs. On the other hand, the photoluminescence intensity also increases with soaking time (Figure 6d and Figure S6b).

**Figure 7:**
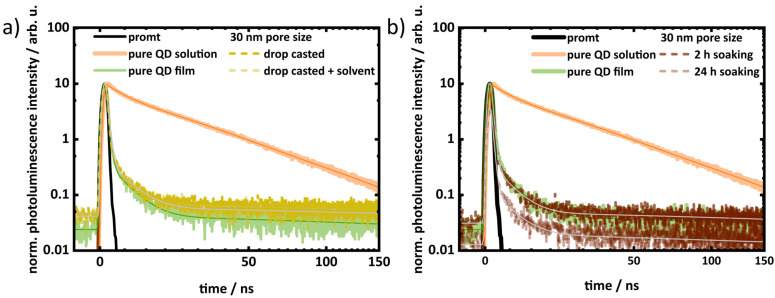
Decay kinetic traces of 30 nm porous layers with QD infiltration via (**a**) drop-casting and (**b**) soaking along with the pure QD both in solution and thin film.

Figure 7 and Figure S7 show the decay traces of pure QDs (in both solution and film) and the treated porous layers. These traces were modeled using a multiexponential function with three components, and the fitted data are presented in Table S1. The average photoluminescence lifetime was determined by amplitude-weighted averaging of the time constants. The pure QD solution has an average lifetime of 38.7 ns, which is reduced to 19.2 ns when deposited on a glass substrate. This reduction is attributed to inter-QD interactions, such as non-radiative energy transfer within layer due to the inhomogeneous QD size distribution [56,57,58]. The photoluminescence decay of QD-infiltrated porous layers is also faster, as shown in Table S1. Porous layers with higher QD loading, as indicated by absorption and photoluminescence spectroscopy, exhibit faster photoluminescence decay. The average photoluminescence lifetime of the 30 nm drop-casted layers is 20.1 ns. However, with the addition of additional solvents, the lifetime decreases to 15.5 ns. Similarly, with increased soaking time, the photoluminescence lifetime of the 30 nm porous layer decreases from 17.8 ns to 11.7 ns. This trend is also observed for the 10 nm porous layers. It is expected that a higher loading of QDs will result in a denser packing inside the pores, leading to a stronger interaction between QDs. Therefore, the faster decay of photoluminescence in porous layers with higher QD loading is attributed to stronger interaction, leading to enhanced non-deactivation. However, there is no significant difference in the photoluminescence lifetime as dependent on pore sizes.

From steady-state absorption and photoluminescence spectroscopy, we can only derive information on the presence of QDs on the substrates in general; it is not possible to distinguish between QDs infiltrated in the pores or just sitting at the surface. An indication of infiltration is that after treating a glass substrate following the soaking method, no QDs are deposited on the substrate (for absorption spectra, see supporting information; Figure S8a). Similar behavior was observed following the drop-casting method, where absorbance at the first excitonic peak was reduced by a factor of six after washing, as depicted in Figure S8b. This indicates that QDs deposited on the surface are removed after the washing step of QD infiltration in porous layers, i.e., the spectra recorded are from the QDs inside pores. 

**Figure 8:**
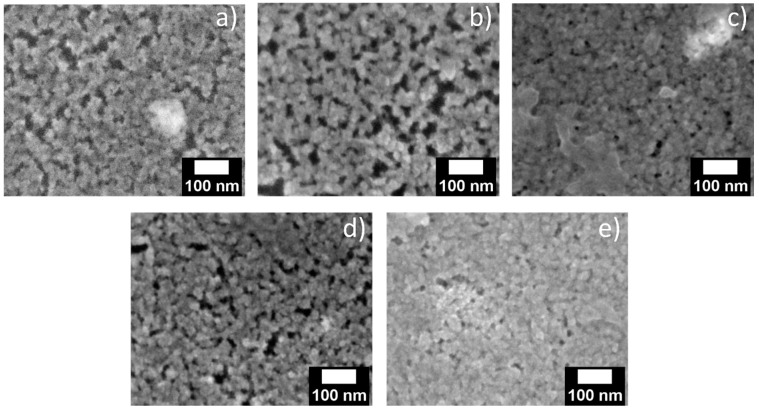
SEM images of (**a**) untreated, (**b**) QD drop-casted, (**c**) QD drop-casted + solvent, (**d**) soaked for 0.5 h and (**e**) soaked for 24 h porous silica layers of 30 nm pore size in silicon wafer.

For further proof for the successful deposition of QDs inside the pores of the porous matrix, scanning electron microscopy (SEM) images were collected for porous layers on silicon substrates treated via the methods described above. Due to the insufficient contrast, it is difficult to localize QDs in the porous matrix in a top-view image. Nevertheless, the opening of the pores at the surface of the structure (Figure 8a) seems to be smaller after infiltration (Figure 8b–e), which could be caused by QD infiltration and filling of the pores. Figure 8b,c compares the drop-casted layer with a drop-casted layer with additional solvent treatment, which indicates an increased pore filling after additional solvent treatment, in agreement with the results from absorption and photoluminescence spectroscopy. On the other hand, porous structures gradually fill with soaking time due to higher infiltration (Figure 8d,e). Similar behavior was observed for 10 nm pore-sized layers (Figure S9). Unfortunately, due to limited spatial resolution of the method, cross-sectional images and EDX elemental mapping could not be collected to directly determine the presence of QDs inside the porous layers. 

**Figure 9:**
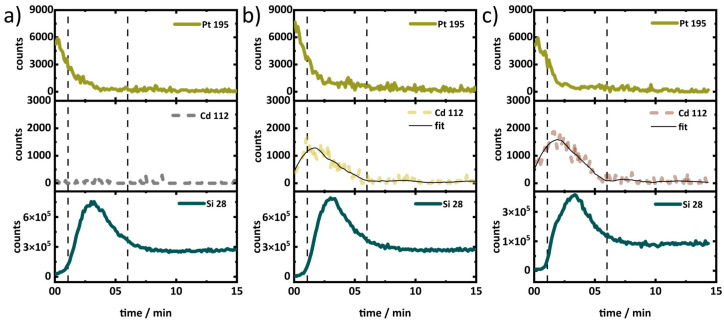
SIMS results of (**a**) drop-casted and untreated, (**b**) drop-casted and solvent treated, and (**c**) soaked for 24 h porous silica layers of 30 nm pore size. The panels from top to bottom correspond to Pt, Cd, and Si concentration. The dashed line at 1 min indicates the position of the top of the porous structure (Pt signal disappears). The second dashed line (6 min) corresponds to the end of porous silica layer, which is determined from the end point of the intense signal of the Si species. After that, the signal of Si is from the non-porous Si wafers.

To collect further proof of the presence of CdSe QDs inside the pores of the porous silica layers, Secondary Ion Mass Spectrometry (SIMS) was performed with a depth profile. A layer of less than 10 nm Platinum (Pt) was deposited on top of the porous layer for electrical non-charging and as an indicator for when the porous layer structure was reached in the sputtering process. The layer system of thin Pt film (for electrical non-charging) on porous layers on the Si wafer were sputtered by an ion beam of the SIMS setup. The secondary ions generated from the material were recorded over the depth. Figure 9 shows the SIMS depth profile of a QD infiltrated porous silica layer of 30 nm pore size. Generated secondary ions from the porous layers were detected with respect to time (plotted in the *X*-axis), which is correlated to the depth of the layer. The region of interest in the SIMS depth profiles is between the two dashed lines, which corresponds to the porous layer. As expected, the untreated porous layer does not contain Cd, which is why no signal is observed (Figure 9a). Figure 9b,c depicts the presence of Cd in the porous layers. Even after washing the porous layers after infiltration, there seems to be small amounts of QDs on the surface, which are seen in the early period (before the dashed line at 1 min) as a less intense Cd signal. Moving towards the depth of the porous layer, the Cd concentration detected increases, which indicates that a higher amount of Cd is present inside the porous layers. The Cd signal levels off with increasing depth, and no signal is detected once the sputtering reaches the Si wafer (dashed line at 6 min). Similar behavior was observed for the layers with 10 nm pore size (see Supplementary Material Figure S10). Hence, the presence of CdSe QDs in the pores is ensured by the SIMS data shown above. The measurement reveals a gradient of deposition of CdSe across the layer and demonstrates that deeper pores may not be reached in the deposition process.

To summarize, we showed the successful infiltration of pre-synthesized CdSe QDs into porous silica thin films with 10 nm and 30 nm pore sizes. It was observed that due to a higher pore volume, 30 nm porous silica shows higher loading after treatment than the 10 nm porous silica matrix. This can be related to a better accessibility of the pores with a higher pore size. The amount of infiltrated QDs can be influenced via drop-casting of additional solvent on a pre-drop-casted porous matrix as well as via varying the soaking time of a porous matrix in a QD solution. Via this method, a porous thin film matrix can be doped with luminescent QDs, which keep their luminescent properties upon deposition. This cannot be achieved via the SILAR process, which lacks controllability over nanocrystal quality and size distribution. 

## Data Availability

The original contributions presented in the study are included in the article/Supplementary Material; further inquiries can be directed to the corresponding author.

## Supplementary Materials

The following supporting information can be downloaded at: https://www.mdpi.com/article/10.3390/ma17174379/s1, Figure S1: Raw absorption spectra of QD infiltrated porous silica layers, fitted scattering curve, and corrected absorption spectra; Figure S2: Uncorrected absorption spectra of untreated porous silica layers of 10 nm and 30 nm pore sizes; Figure S3: 30 nm porous layer with (a) Top-view, (b) cross-section SEM images and 10 nm porous layers with (c) Top-view, and (d) cross-section SEM images; Figure S4: SEM images of (a) untreated porous silica layers of 10 nm pore size in silicon wafer and with (b) 5, (c) 10, (d) 15, and (e) 20 SILAR immersions; Figure S5: (a) TEM image and (b) size distribution determined from TEM image analysis of CdSe QDs. The average size is determined to be 3.2 ± 0.5 nm with an FWHM of 0.5 nm; Figure S6: Absorption spectra (a) and photoluminescence spectra (b) of 10 nm (solid lines) and 30 nm (dashed line) porous layers after 0.5 h and 2 h soaking in a QD solution; Figure S7: Decay kinetic traces of 10 nm porous layers with QD infiltration via (a) drop-casting and (b) soaking along with the pure QD both in solution and thin film; Figure S8: Absorption spectra of glass substrate (a) soaked in a solution of pre-synthesized CdSe QD. ‘Soaking: before washing’ was recorded after removing the substrate from the QD solution, and ‘soaking: after washing’ was recorded after washing the substrate removed from QD solution. Absorption spectra of glass substrate (b) drop-casted with pre-synthesized CdSe QD. ‘Drop casting: before washing’ was recorded after drop-casting of QDs, and ‘drop casting: after washing’ was recorded after washing the substrate drop-casted with QDs; Figure S9: SEM images of (a) untreated, (b) QD drop-casted, (c) QD drop-casted + solvent, (d) soaked for 0.5 h, and (e) soaked for 24 h porous silica layers of 10 nm pore size in silicon wafer; Figure S10: SIMS depth profile of the Pt, Cd, and Si concentration of (a) untreated, (b) drop-casted + solvent, and (c) soaked for 24 h QD in porous silica layers of 10 nm pore size; Figure S11: XRD pattern of pre-synthesized CdSe QD drop-casted on silicon wafer; Table S1: Fitting parameters obtained from triexponential fitting. *τ* and *A* are the time component and relative amplitude. *τ_av_* is the average lifetime. Time components are presented in nanoseconds, ‘ns’.
