# Supplementary material for: Deposition of CdSe Nanocrystals in Highly Porous SiO2 Matrices—In Situ Growth vs. Infiltration Methods
Source: Materials (Basel). 2024 Sep 5;17(17):4379. doi: 10.3390/ma17174379 (PMC11396508; doi:10.3390/ma17174379)
Supplement: Supplementary file 1 [file materials-17-04379-s001.zip › materials-3178892-supplementary.pdf]

Supporting Information

Correction for scattering background of porous layer absorption spectra

To remove the scattering contribution in the collected absorption spectra, a theoretical wavelength dependent scattering function is applied as follows:

$$S = A \times \lambda^{n}$$

This function fitted in the absorption spectra, in the region where there is no absorption of QDs. Here, the wavelength λ is in nm, and the exponent n has values from −4 (Rayleigh scattering for spherical and small particles) to −1 (for larger and non-spherical particles). A represents the background contribution to the absorption spectra [1].

After fitting the scattering function, it was extrapolated to the whole wavelength range, which was then subtracted from the original absorption spectra to obtain the corrected absorption spectra.

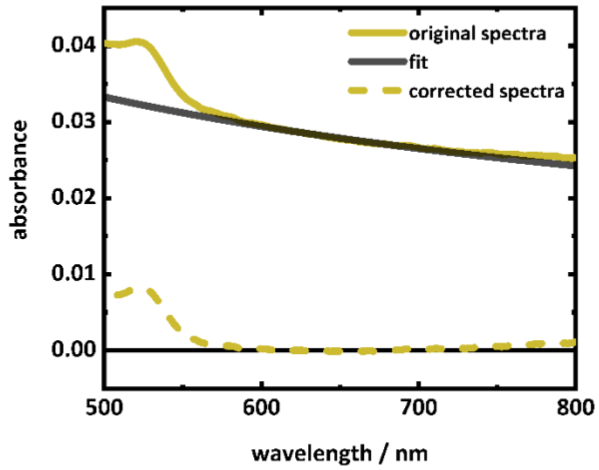

Figure S1. Raw absorption spectra of QD infiltrated porous silica layers, fitted scattering curve, and corrected absorption spectra.

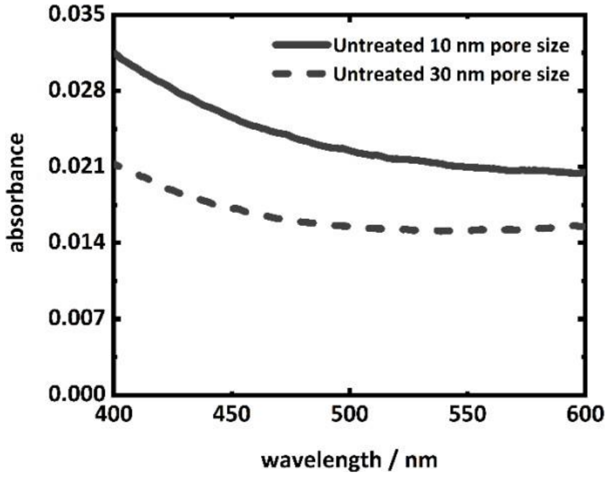

Figure S2. Uncorrected absorption spectra of untreated porous silica layers of 10 nm and 30 nm pore sizes.

Porous silica layers

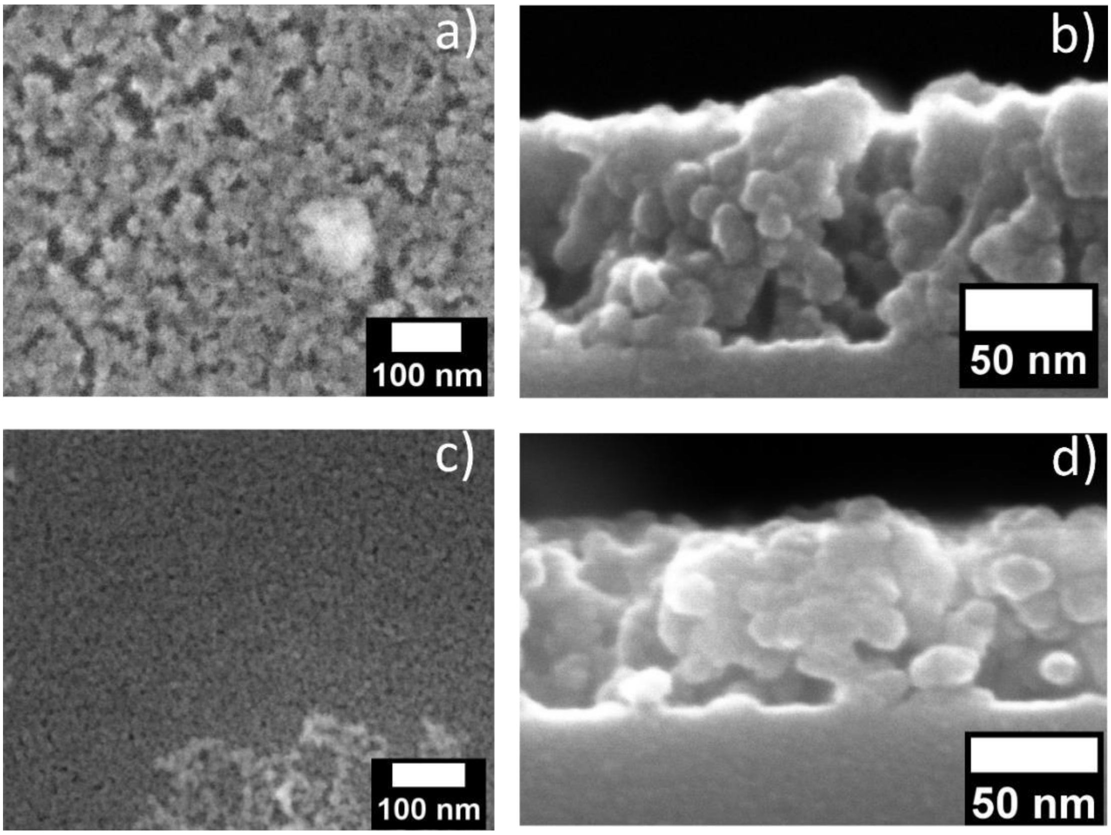

Figure S3. 30 nm porous layer with (**a**) Top-view, (**b**)cross-section SEM images and 10 nm porous layers with (**c**) Top-view, and (**d**) cross-section SEM images.

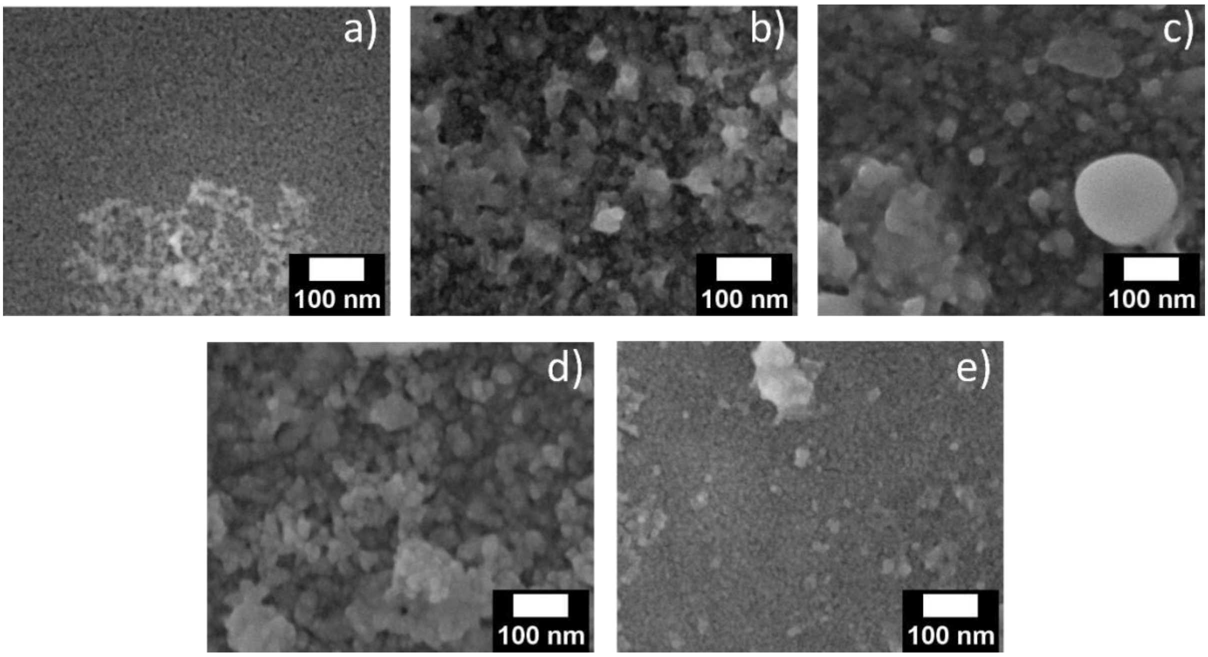

Figure S4. SEM images of (**a**) untreated porous silica layers of 10 nm pore size in silicon wafer and with (**b**) 5, (**c**) 10, (**d**) 15, and (**e**) 20 SILAR immersions.

Size distribution colloidal quantum dots

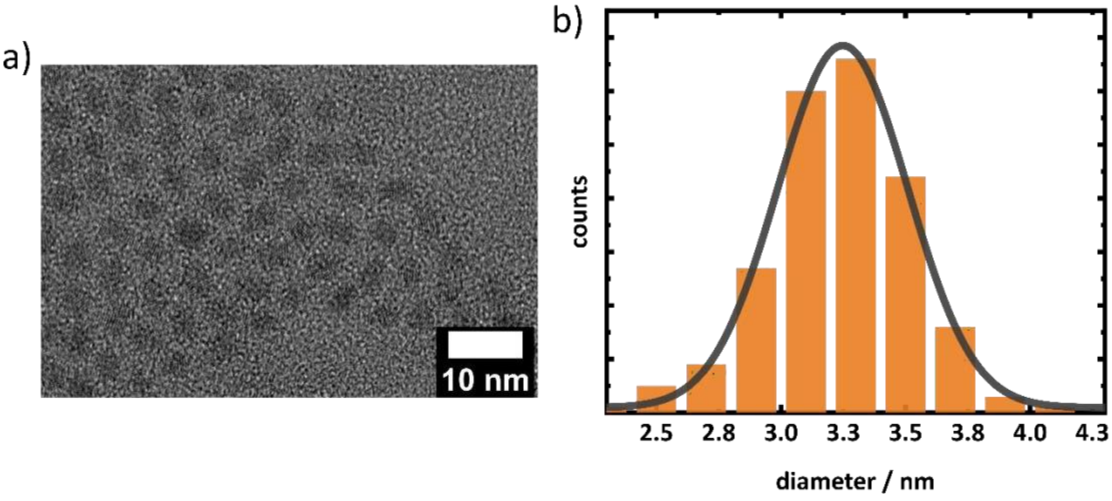

Figure S5. (**a**) TEM image and (**b**) size distribution determined from TEM image analysis of CdSe QDs. The average size is determined to be 3.2±0.5 nm with an FWHM of 0.5 nm.

Absorption spectra and photoluminescence spectra of samples treated by soaking

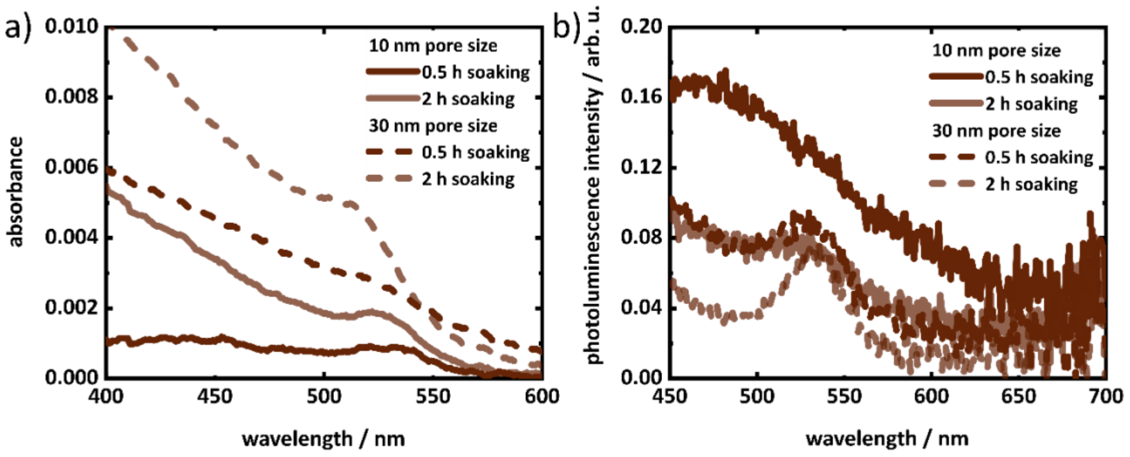

Figure S6. Absorption spectra (**a**) and photoluminescence spectra (**b**) of 10 nm (solid lines) and 30 nm (dashed line) porous layers after 0.5 h and 2 h soaking in a QD solution.

TCSPC data analysis

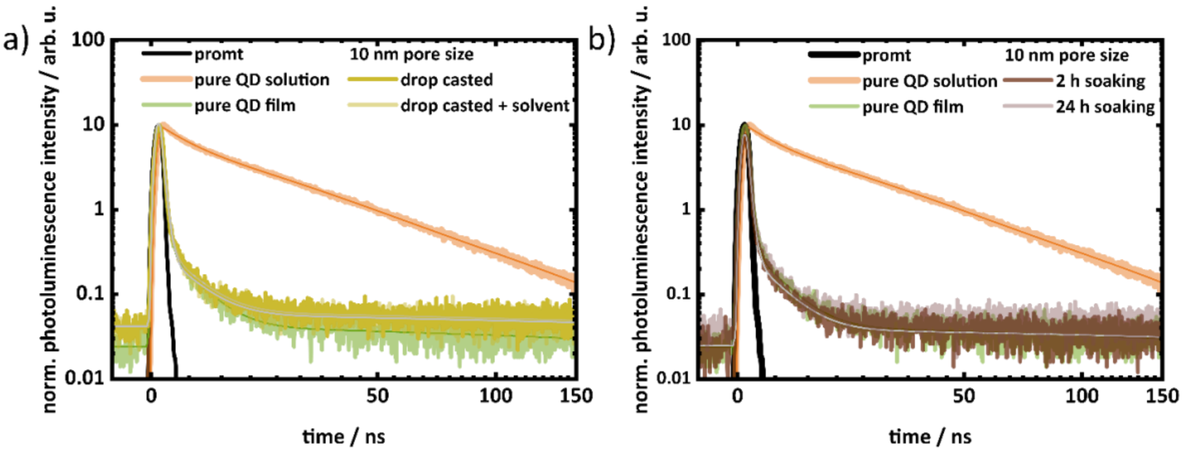

Figure S7. Decay kinetic traces of 10 nm porous layers with QD infiltration via (**a**) drop-casting and (**b**) soaking along with the pure QD both in solution and thin film.

The decay curves were fitted by applying a tri-exponential function:

$$y = y_0 + A_1 e^{-x/\tau_1} + A_2 e^{-x/\tau_2} + A_3 e^{-x/\tau_3}$$

with τ_1, τ_2 and τ_3 being the time components, and A_1, A_2 and A_3 being the pre-exponential factors representing the respective relative amplitudes. The amplitude average lifetime, τ_{av}, was determined according to the equation below.

$$\tau_{av} = \frac{\sum_{i=1}^{n} A_i \tau_i}{\sum_{i=1}^{n} A_i}$$

Fitted parameters obtained from the tri-exponential fitting and the respective average lifetime of all the samples are tabulated below.

Table S1. Fitting parameters obtained from triexponential fitting. τ and A are the time component and relative amplitude. τ_{av} is the average lifetime. Time components are presented in nanoseconds, 'ns'.

Sample		τ_1	τ_2	τ_3	A_1	A_2	A_3	τ_{av}
Pure QD solution		4.2±0.08	20.4±0.52	70.7±0.7	10.3±0.1	50.1±0.2	39.6±0.1	**38.7**±0.5
Pure QD film		0.4±0.004	4.6±0.2	138.6±16.1	77.3±0.3	9.4±0.2	13.3±0.6	**19.2**±2.9
10 nm pore size	drop-casted	0.4±0.005	4.7±0.2	138.1±17.4	76.5±0.3	8.9±0.2	14.6±0.7	**20.9**±3.4
	drop-casted + solvent	0.5±0.004	4.8±0.2	121.6±31.7	80.9±0.3	8.03±0.2	11.03±0.6	**14.1**±4.2
30 nm	drop-casted	0.4±0.005	4.3±0.2	131.7±18.0	75.3±0.3	10±0.2	14.2±0.8	**20.1**±3.5

pore size	drop -casted + solvent	0.4±0.005	4.8±0.2	123.4±28.1	77.4±0.3	10.8±0.2	11.9±0.8	**15.5**±4.3
10 nm pore size	2 h soaking	0.4±0.005	4.7±0.2	196.5±32.9	72.4±0.4	9.5±0.2	18±0.1	**36.2**±7.9
	24 h soaking	0.4±0.005	4.3±0.2	146.9±31.8	77.2±0.3	9.7±0.2	13.1±0.6	**20**±5.2
30 nm pore size	2 h soaking	0.4±0.005	4.3±0.2	132.7±26.6	78.6±0.3	8.5±0.2	12.9±0.7	**17.8**±4.3
	24 h soaking	0.4±0.005	4.3±0.2	146.9±31.8	77.2±0.3	9.7±0.2	13.1±0.6	**11.7**±4.7

QD deposition on glass substrate

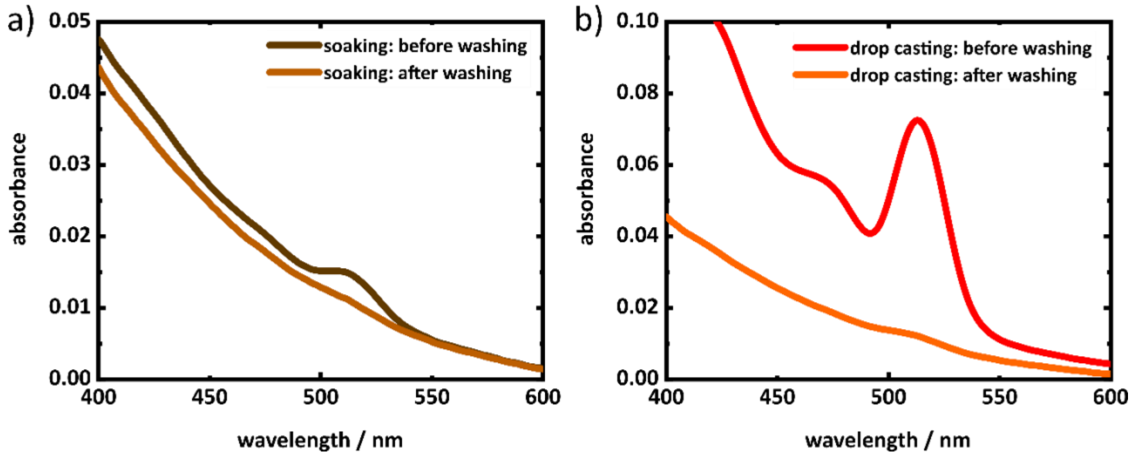

Figure S8. Absorption spectra of glass substrate (**a**) soaked in a solution of pre-synthesized CdSe QD. 'Soaking: before washing' was recorded after removing the substrate from the QD solution, and 'soaking: after washing' was recorded after washing the substrate removed from QD solution. Absorption spectra of glass substrate (**b**) drop-casted with pre-synthesized CdSe QD. 'Drop casting: before washing' was recorded after drop-casting of QDs, and 'drop casting: after washing' was recorded after washing the substrate drop-casted with QDs.

SEM images of treated layers

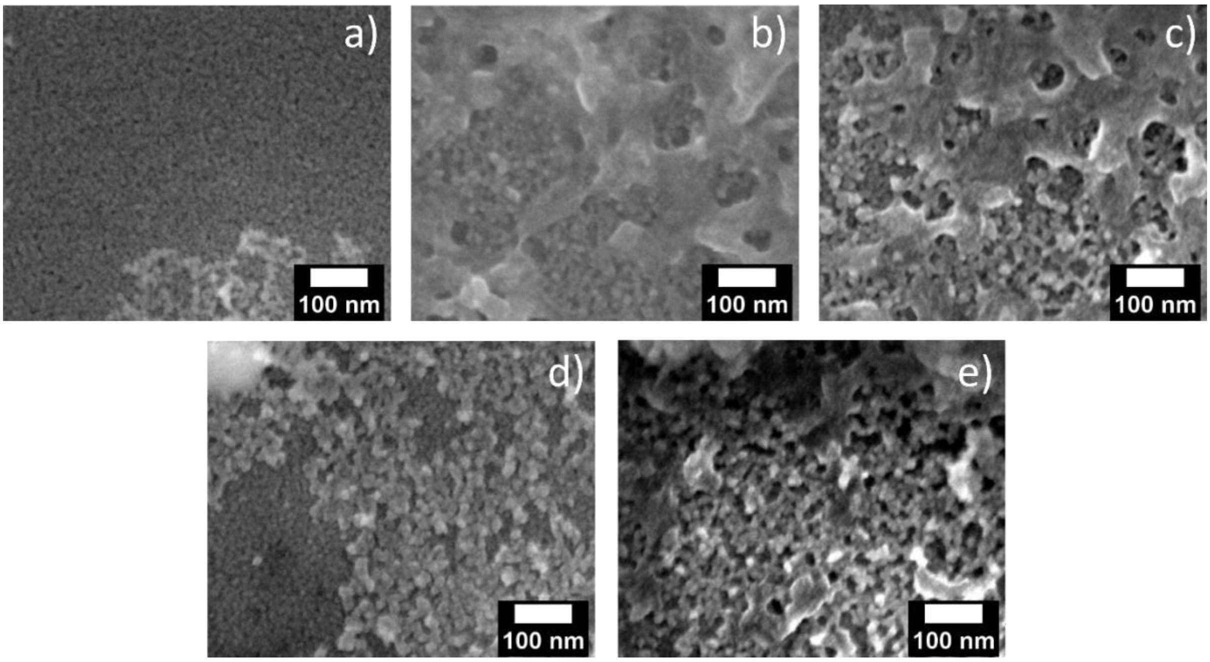

Figure S9. SEM images of (**a**) untreated, (**b**) QD drop-casted, (**c**) QD drop-casted + solvent, (**d**) soaked for 0.5 h, and (**e**) soaked for 24 h porous silica layers of 10 nm pore size in silicon wafer.

SIMS measurements

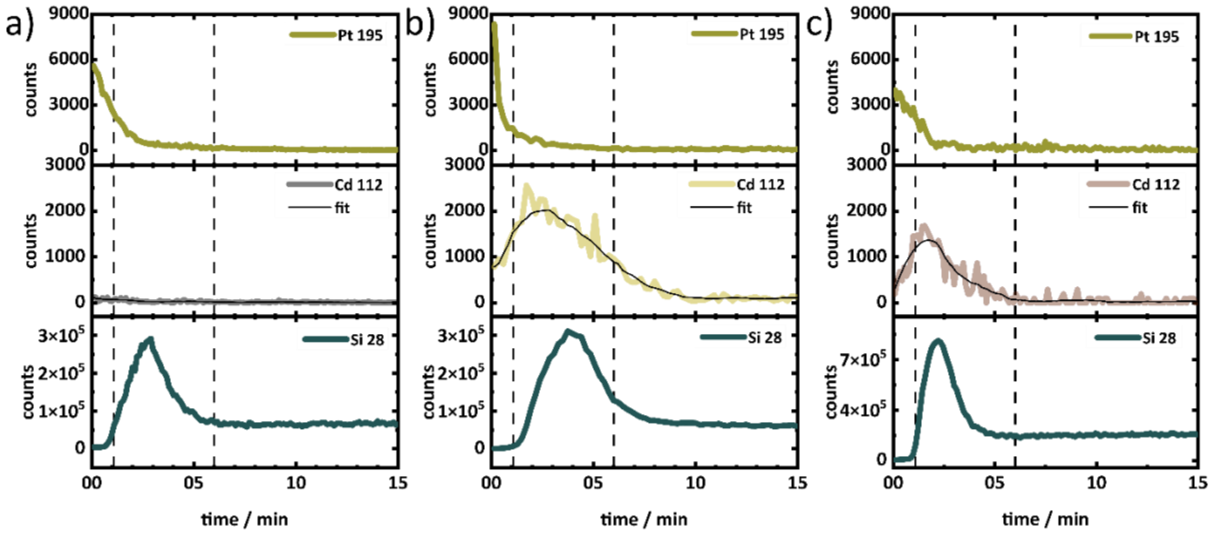

Figure S10. SIMS depth profile of the Pt, Cd, and Si concentration of (**a**) untreated, (**b**) drop-casted + solvent, and (**c**) soaked for 24 h QD in porous silica layers of 10 nm pore size.

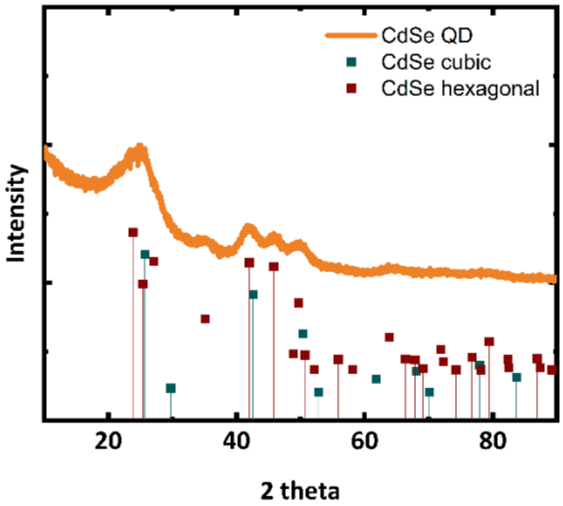

Figure S11. XRD pattern of pre-synthesized CdSe QD drop-casted on silicon wafer.

References

1. Geissler, D.; Wurth, C.; Wolter, C.; Weller, H.; Resch-Genger, U. Excitation wavelength dependence of the photoluminescence quantum yield and decay behavior of CdSe/CdS quantum dot/quantum rods with different aspect ratios. *Phys. Chem. Chem. Phys.* **2017**, *19*, 12509-12516.
